# Author Correction: The *Wolbachia* mobilome in *Culex pipiens* includes a putative plasmid

**DOI:** 10.1038/s41467-019-11234-5

**Published:** 2019-07-12

**Authors:** Julie Reveillaud, Sarah R. Bordenstein, Corinne Cruaud, Alon Shaiber, Özcan C. Esen, Mylène Weill, Patrick Makoundou, Karen Lolans, Andrea R. Watson, Ignace Rakotoarivony, Seth R. Bordenstein, A. Murat Eren

**Affiliations:** 1 ASTRE, INRA, CIRAD, University of Montpellier, Montpellier, 34398 France; 2 Department of Biological Sciences, Vanderbilt University, Nashville, 37235 TN USA; 3 Commissariat à l’Energie Atomique et aux Energies Alternatives (CEA), Institut de Biologie François Jacob, Genoscope, Evry, 91057 France; 4 Graduate Program in the Biophysical Sciences, University of Chicago, Chicago, IL 60637 USA; 5 Department of Medicine, University of Chicago, Chicago, 60637 IL USA; 6 Institut des Sciences de l’Evolution de Montpellier (ISEM), UMR CNRS-IRD-EPHE-Université de Montpellier, Montpellier, 34095 France; 7 Department of Pathology, Microbiology, and Immunology, Vanderbilt University, Nashville, 37235 TN USA; 8 Vanderbilt Institute for Infection, Immunology, and Inflammation, Vanderbilt University, Nashville, 37235 TN USA; 9 Josephine Bay Paul Center for Comparative Molecular Biology and Evolution, Marine Biological Laboratory, Woods Hole, 02543 MA USA

**Keywords:** Mobile elements, Metagenomics

Correction to: *Nature Communications* 10.1038/s41467-019-08973-w, published online 5 March 2019.

The original version of this Article contained an error in Fig. 1a, in which the sequences of the reverse and forward primers were swapped. This has been corrected in both the PDF and HTML versions of the Article.

