# Supplementary material for: The Wolbachia mobilome in Culex pipiens includes a putative plasmid
Source: Nat Commun. 2019 Mar 5;10:1051. doi: 10.1038/s41467-019-08973-w (PMC6401122; doi:10.1038/s41467-019-08973-w)
Supplement: Supplementary file 1 — Supplementary Information [file 41467_2019_8973_MOESM1_ESM.pdf]

Supplementary Information for “The Wolbachia mobilome in *Culex pipiens* includes a putative plasmid” by Reveillaud et al.

Supplementary Note 1. pWCP is widely distributed in *Culex*. We mapped available raw reads from *Wolbachia* metagenome projects to the newly discovered pWCP from O11 in order to investigate the presence of the element in other organisms. We were able to detect the occurrence of pWCP in three different *Culex pipiens* metagenomic libraries from[1] (SRR5810516, Pipiens_MGx_Istanbul; SRR5810518, Pipiens_MGx_Tunis; SRR5810517 Pipiens_MGx_Harash) (Supplementary Figure 1). However, we could not find any evidence of the element in the metagenomes from the fly *Drosophila melanogaster* (SRR018517), the planthoper *Laodelphax striatella* (SRR5179421), and *Anopheles gambiae* mosquito species (SRR1238105 and SRR1238106). It could indicate that *Wolbachia* endosymbionts associated with these different hosts either do not carry plasmids or they carry plasmids that are too divergent from pWCP and therefore do not recruit any reads from the metagenomes.

Further, in order to estimate the coverage of the plasmid as compared to phage and bacterial chromosome genes in the different *Culex pipiens* individuals, we mapped raw reads from the four *Culex pipiens* metagenomes from this study and the three from[1] to the reconstructed MAG O07. MAG O07 was selected because it showed the greatest number of contigs and largest genome size (Supplementary Figure 3, Table 1). While there were as many copies of plasmid-like elements as there were of some *Wolbachia* prophage-associated genes in all four individual mosquitoes from this study, this was not the case in the three additional pooled *Culex pipiens* metagenomic egg samples from[1]. These data suggest the plasmid-like element replication is not coordinated with the phage and that these mobile genetic elements are rather replicating independently. In addition, we observed a high diversity of phage genes in the four samples from this study uniquely, as described earlier (right portion of the graph, Supplementary Figure 3).

Supplementary Note 2. pWCP and and *Wolbachia* bacterial chromosome display similar tetranucleotide composition. Plasmids, which are broadly defined as extranuclear hereditary elements[2], are expected to match the sequence signatures of the host genome as they use the host's replication machinery and share a long history with it[3,4]. The GC content of pWCP (35.52%) indeed resembles that of *Wolbachia*

genomes (33.78 to 33.95%), but to test whether the sequence signatures of pWCP showed similarity to the *Wolbachia* bacterial chromosome at higher levels of resolution, we calculated tetranucleotide frequency (TNF) values for (1) pWCP, (2) each 10 kbp piece of our O11 *Wolbachia* genome, (3) *R. monacensis* genome (Baldridge et al., 2007), and (4) its plasmid, pRM. Organization of each contig based on their TNF signatures revealed two main clusters, one with *R. monacensis* genome and its plasmid, and the other with the *Wolbachia* genome and pWCP (Supplementary Figure 4). The similarity between pWCP TNF signatures and the *Wolbachia* genome supports the hypothesis that pWCP shares an evolutionary history with its *Wolbachia* host and potentially uses *Wolbachia* cellular machinery for expression and replication.

Supplementary Note 3. High sequence divergence between plasmid-like and bacterial gene copies. We identified 11 DnaB gene copies (COG 0305), which clustered in three gene clusters GC_00000322, GC_00001056, and GC_00001060. The two last gene clusters showed plasmid-like characteristics (as indicated by their associated contigs and coverage values ranging from ca. 700 to 2000X) and were shared by the MAGs reconstructed herein. GC_00000322 had coverage values of ca. 150 to 440X, which indicates these are likely bacterial copies of the genes. It was found in all five genomes included in the pangenomic study, that is the four MAGs and *w*Pip Pel. Protein sequences from plasmid-like GC_00001056 and GC_00001060 could not be aligned as they were too divergent, possibly reflecting different regions of the proteins. However, protein sequences from GC_00001056 (plasmid-like) and GC_00000322 (bacteria-like) showed 54.5% divergence at the AA. We observed 100% sequence identity within each gene cluster, showing no sequence difference at the AA level between the plasmid DnaB copies from the wild *Culex pipiens* individuals (GC_00001056 or GC_00001060) as well as between the bacterial DnaB copies from the studied individuals and *w*Pip Pel (GC_00000322). Similarly, we observed 13 mRNA-degrading endonuclease RelE, the toxin component of the RelBE toxin-antitoxin system gene copies (COG 2026) clustering in two plasmid-like gene clusters (GC_00001047 and GC_00001049) only found in the MAGs reconstructed herein and two bacterial-like

gene clusters (GC_00000954 and GC_00001073) shared between wild *Culex pipiens* individuals and the reference genome *w*Pip Pel. Sequences within each gene cluster showed 100% similarity, indicating high sequence conservation of plasmid and bacterial genes, respectively. Gene clusters showed between 51 to 61% sequence divergence between each other. Finally, we identified 8 ParA-like gene copies, which showed similar patterns. Overall, data showed gene function conservation between the extrachromosomal element and the bacterial chromosome such as chromosome partitioning ParA, RelBE, and DnaB yet they exhibited marked diversity at the AA sequence level (more than 50% amino acid sequences divergence), without any evidence to suggest whether the plasmid transmitted these genes to the bacterial chromosome, or vice versa.

Supplementary Figures

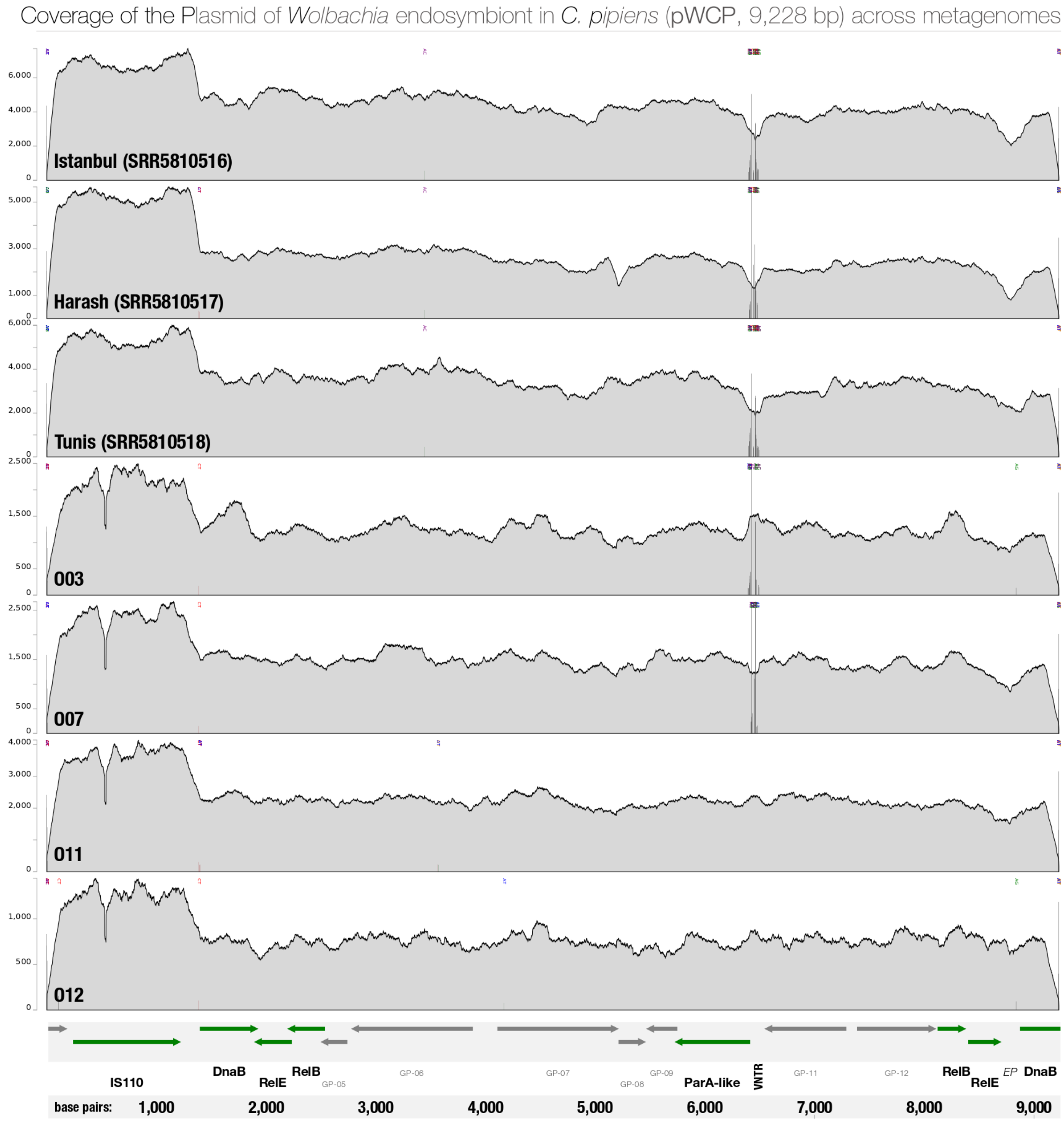

Supplementary Figure 1. Mapping of reads on O11 pWCP in each of the four *Culex pipiens* individuals from this study and the three from[1].

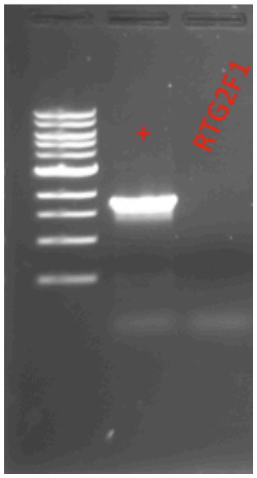

Supplementary Figure 2. PCR amplification of an approximately 1.800 bp sequence using 263F/ 2127 R primers for the untreated *C. pipiens* sample (positive control) and absence of amplification for the *Wolbachia*-free *C. pipiens* sample (RTG2F1, antibiotic Rifampicin treated Generation 2 Female 1).

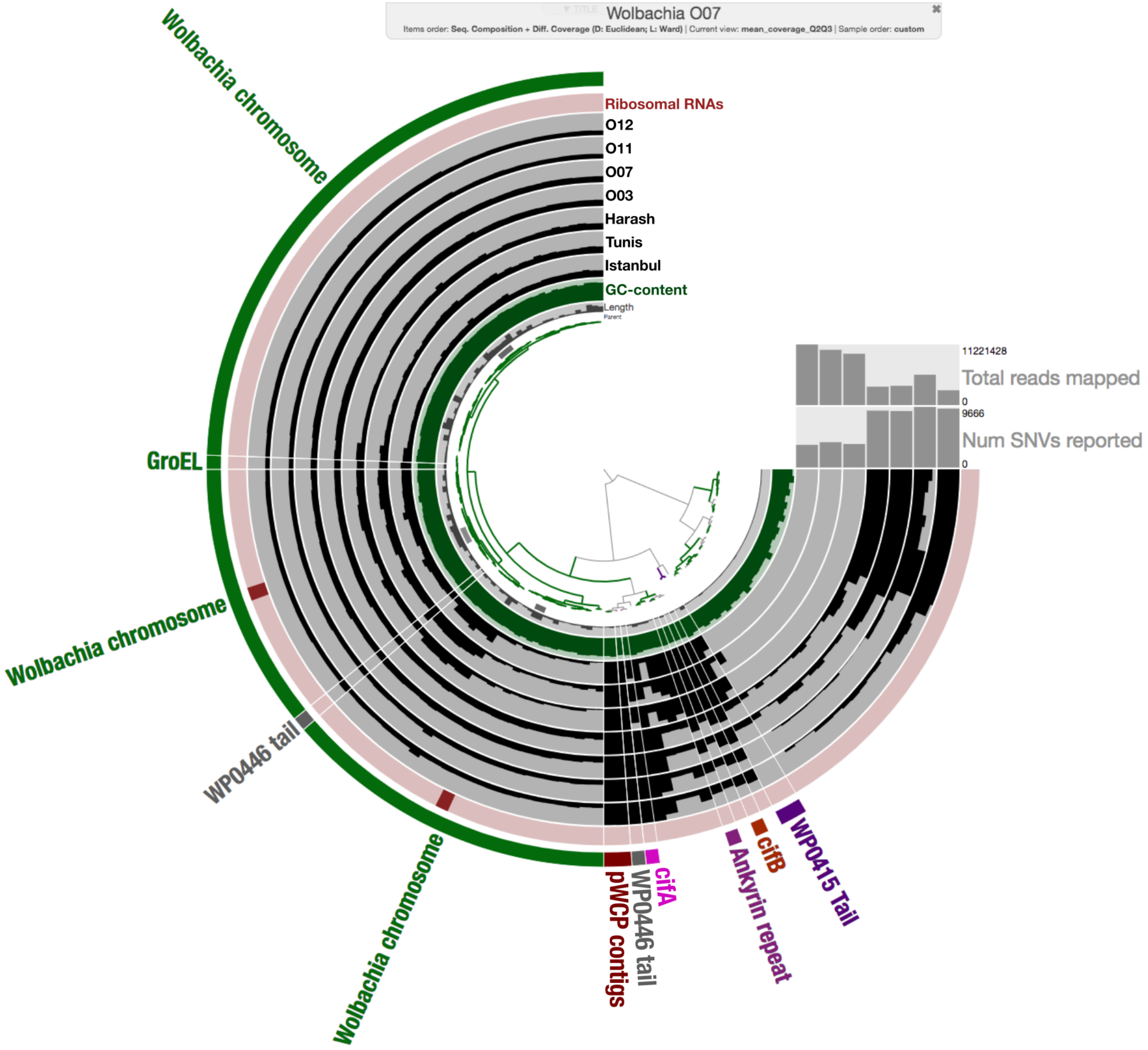

Supplementary Figure 3: Changes of coverage throughout the newly reconstructed metagenome-assembled *Wolbachia* genome in the four *Culex pipiens* metagenomes from this study and the three from Bonneau et al., 2018. The inner clustering dendrogram displays the hierarchical clustering of MAG O07 contigs based on their sequence composition, and their distribution across samples (i.e., differential coverage). Contigs larger than 40 kbp are split into sections of 20 kbp for visualization purposes and each tip on this hierarchical clustering represents a split. Auxiliary layers from inside to outside report information about contigs stored in the contig database (parent marks splits that originate from the same contigs with gray bars, length shows the actual length of a given split, followed by GC-content). In the seven following layers, each bar represents the portion of contigs covered by short reads in a given sample. A complete lack of bar indicates absence of the contig in that sample. The outermost layer shows our selection of key contigs (*Wolbachia* chromosome, plasmid and key phage genes) for which coverage is extracted and reported in Supplementary Table 12. Remaining contigs correspond to contigs carrying phage genes.

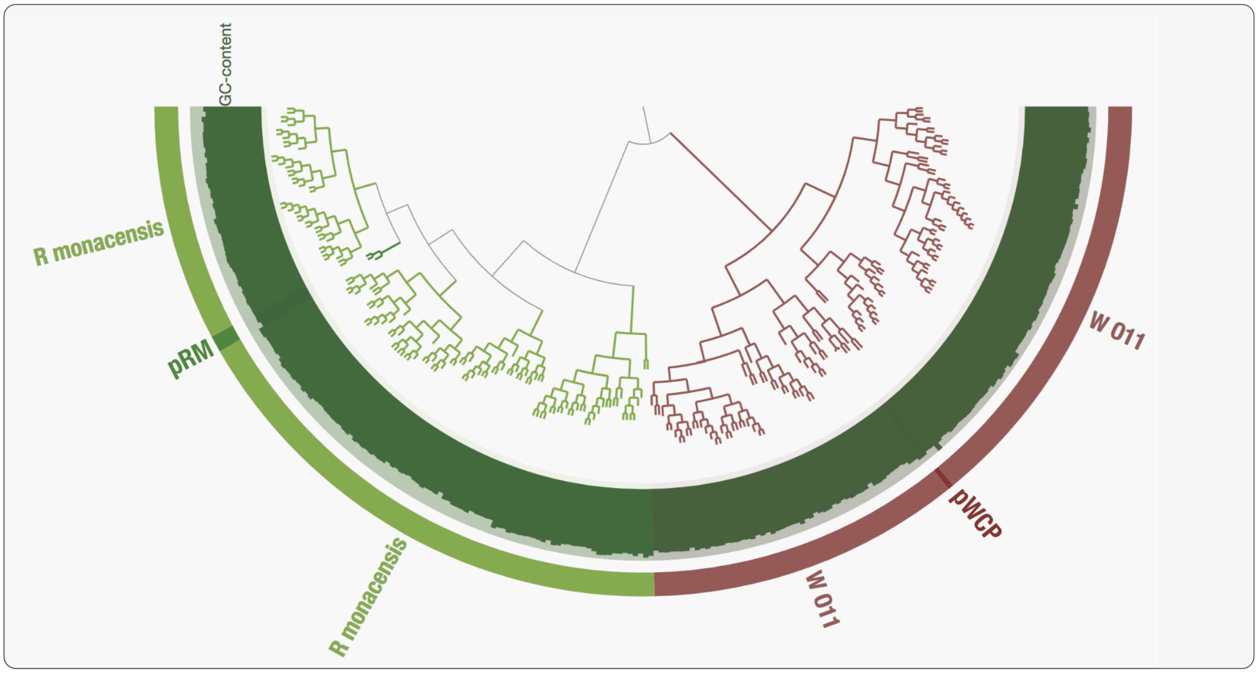

Supplementary Figure 4: Dendrogram of tetranucleotide frequency values support a shared evolutionary history between each bacterial genome and its plasmid. Tetranucleotide frequencies were independently calculated for each 10,000 nt long piece of *R. monacensis* genome and *R. monacensis*-associated plasmid, pRM, (green) as well as O11 *Wolbachia* genome and *Wolbachia*-associated plasmid, pWCP (red) using Euclidian distance metric and Ward clustering algorithm within anvi'o.

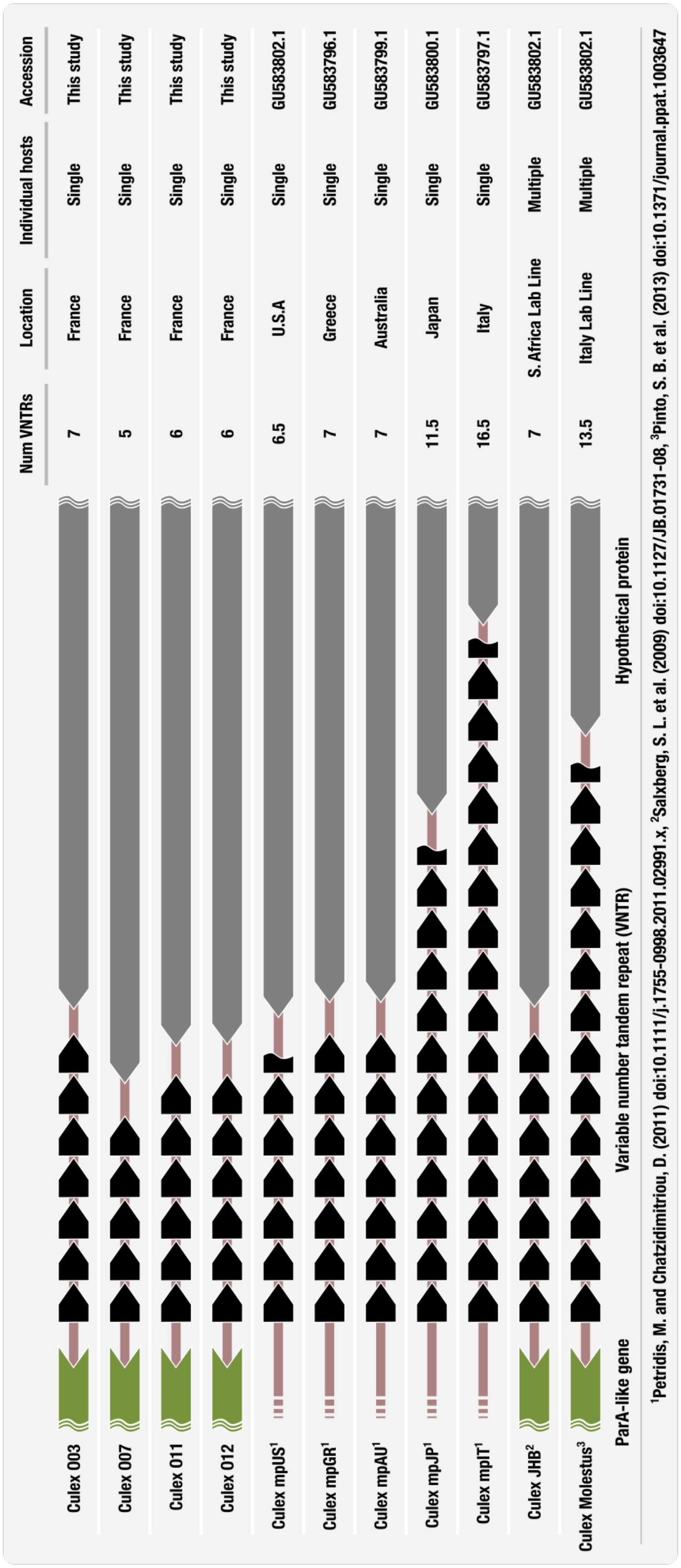

Supplementary Figure 5. Comparison of VNTR region identified in *Culex pipiens* individuals from this study to all available sequences and references. Each black arrow within the VNTR region represents a 16-nt repeat.

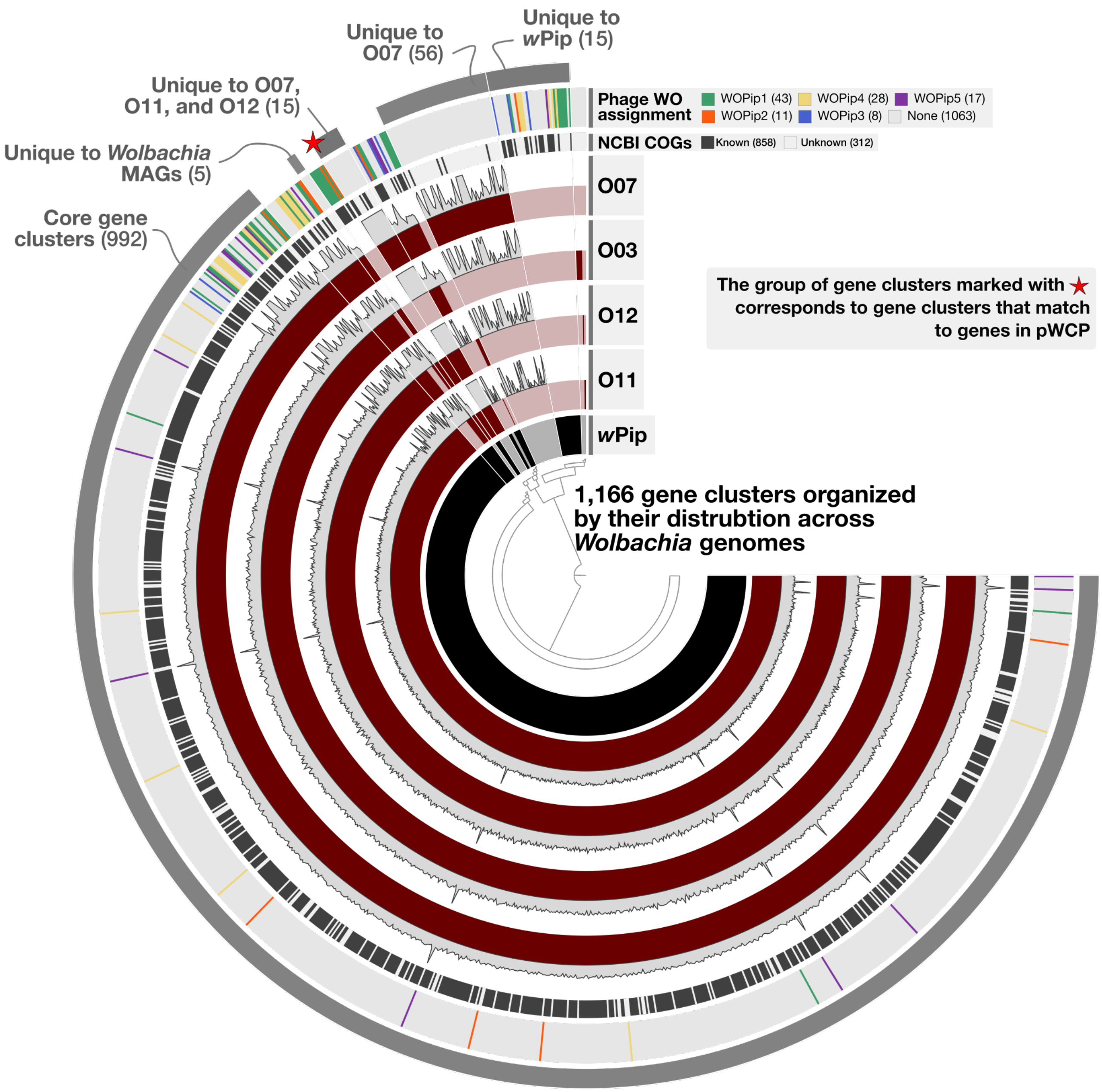

Supplementary Figure 6. *Wolbachia* pangenome; distribution of gene clusters (GC) in the *w*Pip Pel genome and the four *Wolbachia* metagenome-assembled genomes (MAGs) studied herein. In this figure, the inner dendrogram organizes the 1,166 GC based on their distribution across the 5 genomes. The black and red radial circles represent *w*Pip Pel and MAGs, respectively. A bar in the genome layer indicates that the genome had at least one gene that appeared in that gene cluster. The four MAGS-associated line graphs represent mean coverage values of GC in individual MAG O07 (chosen as the reference MAG for read recruitment analysis as it had the highest gene cluster number of all MAGs). The following radial circles represent COGs annotations, and GC assigned to prophage WO (extracted from Klasson et al., 2008). Gene clusters that contain plasmid genes are marked with a star.

Supplementary Tables

	Number of paired-end reads analyzed	Number of high-quality paired-end reads	Number of assembled contigs > 1kbp	O3 Reads mapping	O7 Reads mapping	O11 Reads mapping	O12 Reads mapping
O3	166,686,240	138,140,660 (82,87%)	169,595	96,905,004	87,231,053	82,330,290	73,608,204
O7	188,019,110	156,704,206 (83,34%)	183,463	77,727,981	114,177,333	84,204,122	75,435,256
O11	178,608,248	146,787,838 (82,18%)	162,937	76,549,848	87,625,368	103,749,952	74,071,283
O12	162,285,556	131,957,510 (81,31%)	147,781	75,034,748	85,739,773	81,223,829	90,093,556

Supplementary Table 1. Number of raw reads, high-quality filtered reads, number of assembled contigs longer than 1kbp, and number of reads recruited by these contigs from each metagenome.

x===
Percent SNV density for each *Wolbachia* genome in metagenomes from which they were recovered, the average coverage of nucleotide positions at which SNVs were observed, and the distribution of SNVs across codon positions
x===
Wolbachia in *Culex*_O03: 0.01% (168 SNVs with an avg. cov. of 419X;
31.0% in 1st,
23.8% in 2nd,
45.2% in 3rd nt pos).

Wolbachia in *Culex*_O07: 0.05% (612 SNVs with an avg. cov. of 519X;
31.7% in 1st,
25.5% in 2nd,
42.8% in 3rd nt pos).

Wolbachia in *Culex*_O11: 0.02% (203 SNVs with an avg. cov. of 835X;
31.0% in 1st,
22.7% in 2nd,
46.3% in 3rd nt pos).

Wolbachia in *Culex*_O12: 0.02% (257 SNVs with an avg. cov. of 303X;
28.4% in 1st,
20.6% in 2nd,
51.0% in 3rd nt pos).

See http://merenlab.org/data/2018_Reveillaud_et_al_Wolbachia for the R script that generates this output

Supplementary Table 2. Single nucleotide variants for each *Wolbachia* MAG.

key	Wolbachia_O03	Wolbachia_O07	Wolbachia_O11	Wolbachia_O12	*w*Pip Pel
Wolbachia_O03	1	0.999528674	0.998474454	0.999046288	0.99786132
Wolbachia_O07	0.999664951	1	0.998853245	0.999166228	0.99781632
Wolbachia_O11	0.999322546	0.999353757	1	0.999783509	0.998506996
Wolbachia_O12	0.99846288	0.998154934	0.998585877	1	0.997244936
wPip Pel	0.992606322	0.993011022	0.992734937	0.991047157	1

Supplementary Table 3. Average nucleotide identity across the different *Wolbachia* MAGs and reference genome *w*Pip Pel.

Contigs enriched with bacterial genes	Mean Cov in O03	Mean Cov in O07	Mean Cov in O11	Mean Cov in O12
O03_contigs	239.4071603	228.9333014	471.2134802	168.8200113
O07_contigs	245.1588324	241.5710631	468.9241119	181.4007432
O07_Bis_contigs	255.2607283	311.1894718	269.444004	242.3483702
O11_contigs	238.6043814	228.2484765	475.9977592	170.9946465
O12_contigs	236.9096879	226.5913078	491.1739546	179.4911456
**Contigs enriched with phage genes**	**Mean Cov in O03**	**Mean Cov in O07**	**Mean Cov in O11**	**Mean Cov in O12**
O03_contigs	703.066861	658.3867956	1274.895584	485.096917
O07_contigs	913.9072745	970.7665843	1507.365744	676.9078307
O11_contigs	643.7918401	625.1568058	1315.345249	477.6879496
O12_contigs	788.9371835	749.9859658	1519.015442	591.8282665

Supplementary Table 4. Average coverage of contigs in bacterial vs. phage contig bins in the different *Culex pipiens* ovary metagenomes. Because a set of bacterial O07 contigs showed slightly different coverage values in each sample, they were binned independently and retained as O07_Bis_Contigs.

Primer name	Sequence (5' - 3')
263F	CTAGAGGCCGCAAAGCTCTT
2127R	CGTCTTGTTCGATGCTCCCT
EC_1F	AGGAGCCTCCGGTTTTAGGT
EC_1R	CAATATTCTGCCGGTCGCAC
EC_2F	CTAGAGGCCGCAAAGCTCTT
EC_2R	CAATTGTTGCCAGCCAGAAGT
EC_3F	GGGACATGTATATGCACCCGT
EC_3R	GAGAGATAGTGCTGTGGCGT
EC_4F*	ACACATCGAGCTAATACCCGT
EC_4R*	CCAAGCTCTGGCATTAAACAGA
EC_5F	GCCGTGAGTTACAACAACGT
EC_5R	TTAAGTTTGTGCGGACGTGC
EC_6F	TGCTAACGCAAGGCTTAAAGAA
EC_6R	CCTGATCCTCTGAGTAGCTGC
EC_7F	GATGGAGCTTCTCTACCAAGGA
EC_7R	GCGCACTGTAACTTTACCGG

Supplementary Table 5. New primers designed from the ends of the plasmid-like contig (263F-2127R) to support circularity, EC_1 through EC_7 to Sanger sequence across the gap, and EC_4F-EC_4R(*) to specifically amplify IS110 TE.

	Culex_JHB	Culex_O11	Culex_O12	Culex_O03	Culex_O07
**Culex_JHB**		99.74%	99.74%	99.94%	99.53%
**Culex_O11**	99.74%		100%	99.79%	99.81%
**Culex_O12**	99.74%	100%		99.79%	99.81%
**Culex_O03**	99.94%	99.79%	99.79%		99.65%
**Culex_O07**	99.53%	99.81%	99.81%	99.65%	

Supplementary Table 6. Nucleotide alignment of circular chromosomes from the four wild-caught *Culex pipiens* individuals and JHB

Supplementary References

1. Bonneau, M. *et al.* Culex pipiens crossing type diversity is governed by an amplified and polymorphic operon of Wolbachia. *Nat. Commun.* **9,** 319 (2018).
2. Lederberg, J. Cell genetics and hereditary symbiosis. *Physiol. Rev.* **32,** 403–430 (1952).
3. Campbell, A., Mrazek, J. & Karlin, S. Genome signature comparisons among prokaryote, plasmid, and mitochondrial DNA. *Proc. Natl. Acad. Sci.* **96,** 9184–9189 (1999).
4. van Passel, M. W. J., Bart, A., Luyf, A. C. M., van Kampen, A. H. C. & van der Ende, A. Compositional discordance between prokaryotic plasmids and host chromosomes. *BMC Genomics* **7,** 1–8 (2006).
