# Supplementary material for: The Wolbachia mobilome in Culex pipiens includes a putative plasmid
Source: Nat Commun. 2019 Mar 5;10:1051. doi: 10.1038/s41467-019-08973-w (PMC6401122; doi:10.1038/s41467-019-08973-w)
Supplement: Supplementary file 3 — Description of Additional Supplementary Files [file 41467_2019_8973_MOESM3_ESM.pdf]

Description of Additional Supplementary Files

File Name: Supplementary Data 1
Description: **Contig's abbreviation and coverage**. Abbreviation of actual contig name from assembly outputs with associated coverage values.

File Name: Supplementary Data 2
Description: **Long read data**. Long read ID, match to pWCP, Best hit on ncbi, coverage (i.e., part of the long-read sequence that aligned to best hit in NCBI), long read length and sequence.

File Name: Supplementary Data 3
Description: **pWCP Annotation**. E-values from NCBI Conserved Domain, SMART, and HHpred (including SCOPe, Pfam, and COG) for each pWCP gene.

File Name: Supplementary Data 4
Description: **Summary of the pangenome**. Each entry describes a single gene where columns represent fields for gene cluster id, bin name, genome name, gene caller's id, number of genomes contributing to the gene clusters, maximum number of genes contributing from a single genome (paralogs) per gene cluster, single copy genes, COG function accession number, COG function, COG category accession number, COG category, and amino acid sequence.

File Name: Supplementary Data 5
Description: **Unique MAG's gene clusters**. List of gene clusters that are only found in MAGs and not in *w*Pip Pel. Each entry describes a single gene where columns represent fields for gene cluster id, bin name, genome name, gene caller's id, number of genomes contributing to the gene clusters, maximum number of genes contributing from a single genome (paralogs) per gene cluster, single copy genes, COG function accession number, COG function, COG category accession number, COG category, and amino acid sequence. Eukaryotic-like viral genes are highlighted in red.

File Name: Supplementary Data 6
Description: **Key gene's coverage values**. Mean coverage of key *Wolbachia* chromosome, phage and plasmid genes in the four *Culex pipiens* individuals from this study and the three from Bonneau et al., 2018 (top). The associated coverage ratio between contig and *Wolbachia* chromosome for each sample is also provided (bottom) where green is low and red is high coverage, respectively.
