# Supplementary material for: The Wolbachia mobilome in Culex pipiens includes a putative plasmid
Source: Nat Commun. 2019 Mar 5;10:1051. doi: 10.1038/s41467-019-08973-w (PMC6401122; doi:10.1038/s41467-019-08973-w)
Supplement: Supplementary file 10 — Reporting Summary [file 41467_2019_8973_MOESM10_ESM.pdf]

nature research

Corresponding author(s): Julie Reveillaud and A. Murat Eren

Last updated by author(s): 2019/02/01

Reporting Summary

Nature Research wishes to improve the reproducibility of the work that we publish. This form provides structure for consistency and transparency in reporting. For further information on Nature Research policies, see Authors & Referees and the Editorial Policy Checklist.

Statistics

For all statistical analyses, confirm that the following items are present in the figure legend, table legend, main text, or Methods section.

n/a	Confirmed	
☒	☐	The exact sample size (*n*) for each experimental group/condition, given as a discrete number and unit of measurement
☒	☐	A statement on whether measurements were taken from distinct samples or whether the same sample was measured repeatedly
☒	☐	The statistical test(s) used AND whether they are one- or two-sided<br>*Only common tests should be described solely by name; describe more complex techniques in the Methods section.*
☒	☐	A description of all covariates tested
☒	☐	A description of any assumptions or corrections, such as tests of normality and adjustment for multiple comparisons
☒	☐	A full description of the statistical parameters including central tendency (e.g. means) or other basic estimates (e.g. regression coefficient) AND variation (e.g. standard deviation) or associated estimates of uncertainty (e.g. confidence intervals)
☒	☐	For null hypothesis testing, the test statistic (e.g. *F*, *t*, *r*) with confidence intervals, effect sizes, degrees of freedom and *P* value noted<br>*Give P values as exact values whenever suitable.*
☒	☐	For Bayesian analysis, information on the choice of priors and Markov chain Monte Carlo settings
☒	☐	For hierarchical and complex designs, identification of the appropriate level for tests and full reporting of outcomes
☒	☐	Estimates of effect sizes (e.g. Cohen's *d*, Pearson's *r*), indicating how they were calculated

Our web collection on statistics for biologists contains articles on many of the points above.

Software and code

Policy information about availability of computer code

Data collection	N\A
Data analysis	For most of our analyses we used the open-source software platform anvi'o v5, which is available from http://merenlab.org/software/anvio. In addition, the URL http://merenlab.org/data/2018_Reveillaud_et_al_Wolbachia gives access to a reproducible bioinformatics workflow document and ad hoc scripts used for remaining computational analyses.

For manuscripts utilizing custom algorithms or software that are central to the research but not yet described in published literature, software must be made available to editors/reviewers. We strongly encourage code deposition in a community repository (e.g. GitHub). See the Nature Research guidelines for submitting code & software for further information.

Data

Policy information about availability of data

All manuscripts must include a data availability statement. This statement should provide the following information, where applicable:

- Accession codes, unique identifiers, or web links for publicly available datasets
- A list of figures that have associated raw data
- A description of any restrictions on data availability

The raw sequencing data for shotgun metagenomes are available in the European Nucleotide Archive via the accession code "PRJEB26028 [https://www.ebi.ac.uk/ena/data/search?query=PRJEB26028]". Sequencing data for the MinION run is available at doi:10.6084/m9.figshare.7306784. We also made available FASTA files for individual metagenomic assemblies (doi:10.6084/m9.figshare.6263867), the four metagenome-assembled Wolbachia genomes (doi:10.6084/m9.figshare.6292040), artificially circularized individual plasmid sequences (doi:10.6084/m9.figshare.6380015), as well as anvi'o merged profile databases (doi:10.6084/m9.figshare.6263876), and anvi'o files for the Wolbachia pangenome (doi:10.6084/m9.figshare.6291650).

Field-specific reporting

Please select the one below that is the best fit for your research. If you are not sure, read the appropriate sections before making your selection.

☐ Life sciences ☐ Behavioural & social sciences ☒ Ecological, evolutionary & environmental sciences

For a reference copy of the document with all sections, see nature.com/documents/nr-reporting-summary-flat.pdf

Ecological, evolutionary & environmental sciences study design

All studies must disclose on these points even when the disclosure is negative.

Study description	We studied four wild Culex pipiens individuals captured in Southern France from a single collect and generated an average 70 million Illumina paired-end sequences from the ovaries of each individual through shotgun metagenomics. Using state-of-the-art assembly and binning strategies, we were able to reconstruct near-complete Wolbachia genomes from each individual.
Research sample	Research sample included a group of Culex pipiens ovaries dissected from individuals captured in Southern France and existing Culex pipiens metagenomes from Turkey, Algeria, and Tunisia extracted from (Bonneau, M. et al. Culex pipiens crossing type diversity is governed by an amplified and polymorphic operon of Wolbachia. Nat. Commun. 9, 319 (2018)) as well as reference Wolbachia genome wPip extracted from (Klasson, L. et al. Genome evolution of Wolbachia strain wPip from the Culex pipiens group. Mol. Biol. Evol. 25, 1877–1887 (2008)). Antibiotic treated Culex pipiens and Culex quinquefasciatus individuals were provided by Animals Plant Health Agency, UK and ISEM, France to PCR test in silico findings.
Sampling strategy	Choice of sample size (4 individuals from the same location and trap) built on previous symbiosis work and experience that allowed to reconstruct genomes and investigate inter-individual diversity.
Data collection	Data were collected by mosquito experts from the Entente Interdépartementale de Démoustication (EID) in Montpellier, France.
Timing and spatial scale	17 May 2017
Data exclusions	No data were excluded from the analysis.
Reproducibility	The URL http://merenlab.org/data/2018_Reveillaud_et_al_Wolbachia gives access to a reproducible bioinformatics workflow document and ad hoc scripts used for all computational analyses.
Randomization	Mosquito individuals were sampled randomly.
Blinding	Data acquisition and analysis was done in a completely blind manner. These were our first bacterial binning and long reads sequencing attempts from mosquito samples.

Did the study involve field work? ☒ Yes ☐ No

Field work, collection and transport

Field conditions	We do not have any specific weather forecast condition (rain) to report.
Location	Vic-la-Gardiole, France (43.491973 3.779693)
Access and import/export	Individuals were collected by expert agents from the Entente Interdépartementale de Démoustication (EID) in France in compliance with local authorities in designated sites.
Disturbance	The data were collected overnight and did not cause any disturbance to local environnement.

Reporting for specific materials, systems and methods

We require information from authors about some types of materials, experimental systems and methods used in many studies. Here, indicate whether each material, system or method listed is relevant to your study. If you are not sure if a list item applies to your research, read the appropriate section before selecting a response.

Materials & experimental systems

n/a	Involved in the study
☒	☐ Antibodies
☒	☐ Eukaryotic cell lines
☒	☐ Palaeontology
☒	☐ Animals and other organisms
☒	☐ Human research participants
☒	☐ Clinical data

Methods

n/a	Involved in the study
☒	☐ ChIP-seq
☒	☐ Flow cytometry
☒	☐ MRI-based neuroimaging
